# Probabilistic Interaction Network of Evidence Algorithm and its Application to Complete Labeling of Peak Lists from Protein NMR Spectroscopy

**DOI:** 10.1371/journal.pcbi.1000307

**Published:** 2009-03-13

**Authors:** Arash Bahrami, Amir H. Assadi, John L. Markley, Hamid R. Eghbalnia

**Affiliations:** 1 National Magnetic Resonance Facility at Madison, Biochemistry Department, University of Wisconsin Madison, Madison, Wisconsin, United States of America; 2 Mathematics Department, University of Wisconsin Madison, Madison, Wisconsin, United States of America; 3 Center for Eukaryotic Structural Genomics, University of Wisconsin Madison, Madison, Wisconsin, United States of America; 4 Graduate Program in Biophysics, University of Wisconsin Madison, Madison, Wisconsin, United States of America; New York Structural Biology Center, United States of America

## Abstract

The process of assigning a finite set of tags or labels to a collection of observations, subject to side conditions, is notable for its computational complexity. This labeling paradigm is of theoretical and practical relevance to a wide range of biological applications, including the analysis of data from DNA microarrays, metabolomics experiments, and biomolecular nuclear magnetic resonance (NMR) spectroscopy. We present a novel algorithm, called Probabilistic Interaction Network of Evidence (PINE), that achieves robust, unsupervised probabilistic labeling of data. The computational core of PINE uses estimates of evidence derived from empirical distributions of previously observed data, along with consistency measures, to drive a fictitious system *M* with Hamiltonian *H* to a quasi-stationary state that produces probabilistic label assignments for relevant subsets of the data. We demonstrate the successful application of PINE to a key task in protein NMR spectroscopy: that of converting peak lists extracted from various NMR experiments into assignments associated with probabilities for their correctness. This application, called PINE-NMR, is available from a freely accessible computer server (http://pine.nmrfam.wisc.edu). The PINE-NMR server accepts as input the sequence of the protein plus user-specified combinations of data corresponding to an extensive list of NMR experiments; it provides as output a probabilistic assignment of NMR signals (chemical shifts) to sequence-specific backbone and aliphatic side chain atoms plus a probabilistic determination of the protein secondary structure. PINE-NMR can accommodate prior information about assignments or stable isotope labeling schemes. As part of the analysis, PINE-NMR identifies, verifies, and rectifies problems related to chemical shift referencing or erroneous input data. PINE-NMR achieves robust and consistent results that have been shown to be effective in subsequent steps of NMR structure determination.

## Author Summary

What mathematicians call the “labeling problem” underlies difficulties in interpreting many classes of complex biological data. To derive valid inferences from multiple, noisy datasets, one must consider all possible combinations of the data to find the solution that best matches the experimental evidence. Exhaustive searches totally outstrip current computer resources, and, as a result, it has been necessary to resort to approximations such as branch and bound or Monte Carlo simulations, which have the disadvantages of being limited to use in separate steps of the analysis and not providing the final results in a probabilistic fashion that allows the quality of the answers to be evaluated. The Probabilistic Interaction Network of Evidence (PINE) algorithm that we present here offers a general solution to this problem. We have demonstrated the usefulness of the PINE approach by applying it to one of the major bottlenecks in NMR spectroscopy. The PINE-NMR server takes as input the sequence of a protein and the peak lists from one or more multidimensional NMR experiments and provides as output a probabilistic assignment of the NMR signals to specific atoms in the protein's covalent structure and a self-consistent probabilistic analysis of the protein's secondary structure.

## Introduction

Labeling a set of fixed data with another representative set is the generic description for a large family of problems. This family includes clustering and dimensionality reduction, an approach in which the original dataset is represented by a set of typically far lower dimension (the representative set). The representative set, often the parameter vector that signifies a set of data points, can be simply the cluster mean (center) or may include additional parameters, such as the cluster diameter. The labeling problem is important, because it is encountered in many applications involving data analysis, particularly where prior knowledge of the probability distributions is incomplete or lacking.

A challenging instance of the labeling problem arises naturally in nuclear magnetic resonance (NMR) spectroscopy, which along with X-ray crystallography is one of the two major methods for determining protein structures. Although NMR spectroscopy is not as highly automated as the more mature X-ray field, it has advantages over X-ray crystallography for structural studies of small proteins that are partially disordered, exist in multiple stable conformations in solution, exhibit weak interactions with ligands, or fail to crystallize readily [1], provided that the NMR signals can be assigned to specific atoms in the covalent structure of the protein. The labeling problem known as the “assignment problem”, has been one of the major bottlenecks in protein NMR spectroscopy.

**Figure 1:**
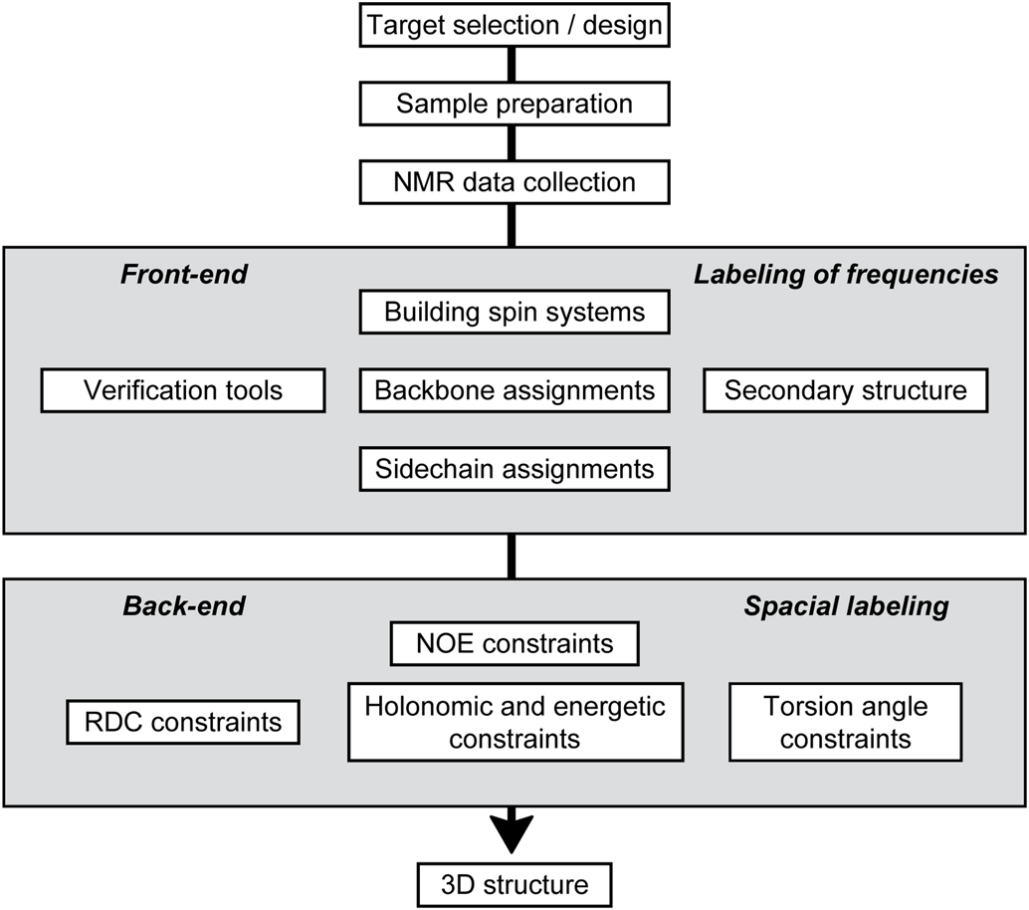
Conventional stages in protein structure determination by NMR. After the data have been collected, the challenging “front-end” process leads to sequence-specific amino acid labeling. The “back-end” process then leads to the three-dimensional structure.

Protein NMR structure determination generally proceeds through a series of steps (Figure 1). The usual approach is first to collect data used in determining backbone and aliphatic side chain assignments (front-end labeling). These assignments are then used to interpret data collected in order to determine interatomic or torsion angular constraints (back-end labeling) used in structure determination.

The front-end “labeling process” associates one or more NMR parameters with a physical entity (e.g., nucleus, residue, tripeptide, helix, chain); the back-end “labeling process” associates NMR parameters with constraints that define or refine conformational states. In reality, the distinction between front-end and back-end is artificial. Strategies have been developed that use NOESY data for assignments [2],[3] or for direct structure determination without assignments [4]. In addition, as demonstrated recently, structures of small proteins can be determined directly from assigned chemical shifts by a process that largely bypasses the back-end [5],[6]. Ideally, all available data should be used in a unified process that yields the best set of assignments and best structure consistent with experiment and with a probabilistic analysis that provides levels of confidence in the assignments and atomic coordinates.

### Prior Approaches to the Problem

**Figure 2:**
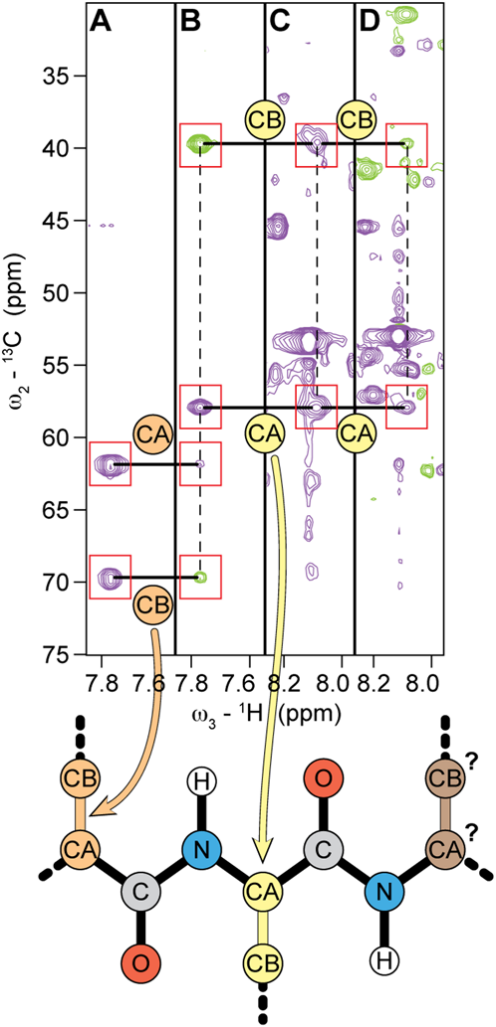
Conventional process of resonance assignments for a protein labeled with stable isotopes (^13^C and ^15^N). Peaks observed in multidimensional spectra are matched to search for common frequencies. Some common frequencies identify atoms within a residue; others identify atoms in neighboring residues. The common visual aid in this process is a series of paired strip plots from complementary NMR experiments. Strips from CBCA(CO)NH (*a* and *c*) and HNCACB (*b* and *d*) experiments can be used here to assign the tripeptide Thr-Tyr-His. Starting with C^α^ (CA) and C^β^ (CB) frequencies assumed to belong to Thr^66^ (strip *a*), a horizontal trace (line), arising from the common frequency of NH nuclei, is used to locate C^α^ and C^β^ of Tyr^67^ in (strip *b*). To continue the process, the same peaks are located in (strip *c*), and the peaks are traced to strip *d*. In strip *d*, given the accepted tolerances across spectra (shown by boxes around the selected peaks), several alternative assignments are plausible for His^68^. These additional peaks may be artifacts (false peaks), or peaks from other nuclei with similar frequency. Depending on the starting point of the assignment process, the choice of experiments, the amount of conflicting information, or other factors, an exponentially expanding number of alternative assignments can arise, rendering a computational solution intractable. This difficulty has proved to be a major drawback for NMR structure determination, particularly for larger proteins.

The usual approach to the solution of the problem of assigning labels to subsets of peaks (spin subsystems) assembled from multiple sets of noisy spectra is to collect a number of multidimensional, multinuclear datasets. After converting the time domain data to frequency domain spectra by Fourier transformation, peaks are picked from each spectrum for analysis. Methods have been developed for automated peak picking or global analysis of spectra to yield models consisting of peaks with known intensity, frequency, phase, and decay rate or linewidth [7],[8]. In the ideal case, the resulting peak-lists identify *combinatorial subsets* of two or more covalently bonded nuclei by their respective frequencies (Figure 2). These subsets must be “assembled” in a coherent way to “best” correspond to specific atoms in the amino acid sequence of the protein. In practice, peak lists do not report on all nuclei (because some peaks are missing), and “noise peaks” (peaks incorrectly reported as true peaks) are commonplace. In the examples analyzed here (Table 1), the level of missing peaks varied between 9% and 38%, while the level of noise peaks varied between 10% and 135%. The large number of false positives as well as false negatives typically present in the data result in an explosion of “ambiguities” during the assembly of subsets.

**Table 1:** Backbone and side chain assignment performance of PINE-NMR with NMR data from a representative group of twelve proteins.

Protein Designator	Number of Residues	Backbone Data Completeness^a^	Correct Backbone Assignment Coverage^b^	Backbone Assignment Accuracy^c^	Side chain Assignment Accuracy^c^	Secondary Structure Accuracy^d^	Outlier Count^e^	Data Quality^f^	Running Time on 1.8Ghz Intel CPU (h)	PISTACHIO Backbone Assignment Accuracy	Level of Missing Peaks in HNCACB Dataset	Noise Level in HNCACB Dataset^g^	Experiments represented in the input peak lists^h^								
													*1*	*2*	*3*	*4*	*5*	*6*	*7*	*8*	*9*
Proteins with both backbone and side chain experiments available:																					
Ubiquitin	76	99%	96%	97%	94%	97%	1	0.91	0.2	91%	12%	20%	X	X				X	X	X	X
Mm202773	101	98%	96%	98%	92%	97%	3	0.92	0.2	97%	24%	42%	X	X				X	X	X	
At1g77540	103	97%	96%	99%	93%	94%	2	0.93	0.2	99%	37%	48%	X	X					X		
At2g24940	109	99%	99%	100%	95%	95%	1	0.91	0.2	100%	29%	10%	X	X				X	X		
At5g22580	111	98%	94%	96%	93%	90%	1	0.89	0.2	92%	21%	17%	X	X				X	X	X	
At3g17210	112	97%	92%	95%	92%	90%	1	0.84	1	90%	9%	14%		X	X	X	X	X	X		
At3g51030	124	96%	92%	96%	91%	88%	2	0.88	1	90%	38%	68%		X	X	X	X	X	X		
At2g46140	174	98%	92%	94%	98%	90%	1	0.86	1	91%	20%	35%	X	X					X	X	X
At3g16450^i^	299	95%	82%	86%	77%	NA	1	0.89	2	80%	23%	135%	X	X	X	X	X	X		X	
Proteins with only backbone experiments available:																					
BMRB5106	70	96%	91%	95%	NA	90%	1	0.88	0.2	90%	10%	25%	X	X							
At2g23090	86	87%	87%	100%	NA	92%	3	0.89	0.2	97%	30%	44%	X	X							
At5g01610	170	96%	81%	84%	NA	83%	3	0.76	1	80%	24%	117%	X	X	X						

a The maximum number of backbone assignment achievable theoretically on the basis of the peak lists provided as input to PINE, divided by the total number of backbone assignments deposited in BMRB, multiplied by 100.

b Number of correct PINE-NMR backbone assignments, divided by the total number of backbone assignments deposited in BMRB, multiplied by 100.

c Number of correct PINE-NMR (backbone/side chain) assignments (i.e. in agreement with those in BMRB), divided by the maximum number of (backbone/side chain) assignments achievable theoretically on the basis the peak lists provided as input to PINE, multiplied by 100.

d Percentage of residues correctly assigned to helix, strand, or “other” by PINE-NMR on the basis of agreement with DSSP [50] analysis of the deposited three-dimensional structure of the protein.

e Total number of C′, C^α^, and C^β^ atoms detected as possible outliers by LACS method [16] in the final assignment.

f Defined as 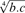 (see Table S1).

g Defined as number of noise peaks divided by number of real peaks in HNCACB.

h All input included data from an HSQC or HNCO experiment; data from additional experiments were as indicated by shaded boxes: *1* CBCA(CO)NH or HN(CO)CACB, *2* HNCACB, *3* HNCA, *4* HN(CO)CA, *5* HN(CA)CO, *6* H(CCO)NH, *7* C(CO)NH, *8* HBHA(CO)NH, *9* HCCH-TOCSY.

i Stereo array isotope labeled (SAIL) protein; data were analyzed without corrections for isotope shifts due to deuterium labeling.

A common feature among prior approaches has been to divide the assignment of labels into a sequence of discrete steps and to apply varying methods at each step. These steps typically include an “assignment step” [9]–[12], a secondary structure determination step [13]–[15], and a “validation step” [16]. The validation step, in which a discrete reliability measure indicates the possible presence of outliers, misassignments, or abnormal backbone chemical shift values, is sometimes omitted. Other steps can be added, or steps can be split further into simpler tasks. For example, backbone and side chain assignments frequently are carried out sequentially as separate processes. Some approaches to sequence-specific assignment rely on a substantially reduced combinatorial set of input data by assuming a prior subset selection, e.g., prior spin system assembly [17],[18]. The specification of conformational states can be added as yet another labeling step. For example, backbone dihedral angles can be specified on a grid (e.g., 30° intervals) as determined from chemical shifts [19], coupling constants and/or NOEs [20], or reduced dipolar couplings [21].

The NMR assignment problem has been highly researched, and is most naturally formulated as a combinatorial optimization problem, which can be subsequently solved using a variety of algorithms. A 2004 review listed on the order of 100 algorithms and software packages [22], and additional approaches are given in a 2008 review [23]. Prior methods have included stochastic approaches, such as simulated annealing/Monte Carlo algorithms [24]–[26], genetic algorithms [27], exhaustive search algorithms [17], [28]–[30], heuristic comparison to predicted chemical shifts derived from homologous proteins [31], heuristic best-first algorithms [32]–[34], and constraint-based expert system that use heuristic best-first mapping algorithm [35]. Of these, the most established, as judged from BMRB entries that cite the assignment software packages used, are Autoassign [10] and GARANT [27].

Similarly, a wide range of methods have been used to predict the protein secondary structural elements that play an important role in classifying proteins [36],[37]. Prior approaches to assigning a secondary structure label to each residue of the protein have included the Δδ method [38], the chemical shift index method [14], a database approach (TALOS) [19], an empirical probability-based method [39], a supervised machine learning approach [40], and a probabilistic approach that utilizes a local statistical potential to combine predictive potentials derived from the sequence and chemical shifts [13]. Recently, a fully automated approach to protein structure determination, FLYA, has been described that pipelines the standard steps from NMR spectra to structure and utilizes GARANT as the assignment engine [41]. The FLYA approach demonstrates the benefits of making use of information from each step in an iterative fashion to achieve a high number of backbone and side chain assignments.

Our goal is to implement a comprehensive approach that utilizes a network model rather than a pipeline model and relies on a probabilistic analysis for the results. We reformulate the combinatorial optimization problem whereby each labeling configuration in the ensemble has an associated but unknown non-vanishing probability. The PINE algorithm enables full integration of information from disparate steps to achieve a probabilistic analysis. The use of probabilities provides the means for sharing and refining incomplete information among the current standard steps, or steps introduced by future developments. In addition, probabilistic analysis deals directly with the multiple minima problem that arises in cases where the data does not support a single optimal and self-consistent state. A common example is a protein that populates two stable conformational states.

The PINE-NMR package described here represents a first step in approaching the goal of a full probabilistic approach to protein NMR spectroscopy. PINE-NMR accepts as input the sequence of the protein plus peak lists derived from one or more NMR experiments chosen by the user from an extensive list of possibilities. PINE-NMR provides as output a probabilistic assignment of backbone and aliphatic side chain chemical shifts and the secondary structure of the protein. At the same time, it identifies, verifies, and, if needed, rectifies, problems related to chemical shift referencing or the consistency of assignments with determined secondary structure. PINE-NMR can make use of prior information derived independently by other means, such as selective labeling patterns or spin system assignments. In principle, the networked model of PINE-NMR is extensible in both directions within the pipeline for protein structure determination (Figure 1): it can be combined with adaptive data collection at the front or with three-dimensional structure determination at the back end. Such extensions should lead to a rapid and fully automated approach to NMR structure determination that would yield the structure most consistent with all available data and with confidence limits on atom positions explicitly represented.

In addition to its application to NMR spectroscopy, the PINE approach should be applicable to the unbiased classification of biological data in other domains of interest, such as systems biology, in which data of various types need to be integrated: genomics (DNA chips), proteomics (MS analysis of proteins), and metabolomics (GC-MS, LC-MS, and NMR) data collected as a function of time and environmental variables.

## Methods

### General Approach

**Figure 3:**
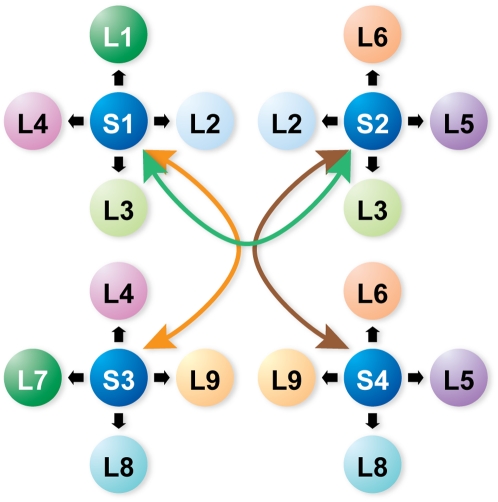
Illustration of the system of neighborhoods built around each data value in PINE. Each input data point (S) is linked to a set of labels (L) with associated weights. Similarity measures and constraints are utilized to construct each neighborhood system or topology (as denoted by the arrows).

The fundamental idea of PINE is to embed the original assignment problem into a higher dimensional setting and to use empirically estimated compatibility (or similarity) conditions to iteratively arrive at an internally coherent labeling state. These conditions are embodied in the form of a parameterized Hamiltonian (energy function) that evolves at each iteration step. In the quasi-stationary regime, this construction yields clusters, defined as subsets of chemical shift data with assigned labels. The clusters have strong intra-cluster links and highly localized inter-cluster couplings. We view each possible cluster of related experimental data in the domain as a “site” that is to be potentially labeled. More specifically, our goal is to discover (learn) the map *f* that relates the “domain” (set of subsets of data) to the “codomain” (set of subsets of labels):
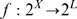
where *X* = [*x*_1_, *x*_2_, …, *x*_m_] is the set of data values available from all experiments, and *L* = [*L*_1_, *L*_2_, …, *L*_n_] is the set of labels associated to the chemical shifts. At first it may appear that this map is trivial, because one protein has precisely one set of correct chemical shifts. However, breaks in the backbone sequential data, incompleteness of 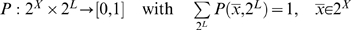 experimental peak lists, and the presence of many noise peaks renders the discovery of a deterministic one-to-one map to the sequential labels unpromising. Rather than discovering a single map, we opt to find a set of maps, each with its associated probability. More directly, we choose to associate subsets of labels from the list *L* to subsets of data from the list *X*, each with a commensurate probability:

In order to formulate the computational problem, we require that the labels for data values satisfy constraints that arise from the system of neighborhoods built around each data value. The system of neighborhoods is a dynamic state variable that co-evolves with the probability values. We assign an initial set of labels, L, with associated weights to each input data point, S (e.g., chemical shift) and introduce a measure of similarity based on distances between “neighboring points” (Figure 3). Typically, in our starting configuration, the possible labels for each data value far exceed the number of sites. The set of labels contains the “*null*” label to allow for the case where a data element cannot be labeled.

The approach used to measure the global compatibility or support for the specific labeling of site *S* at iteration step *m* is to aggregate the compatibilities over versions of individual evidences by applying a variation of the belief propagation algorithm [42]. The evidence for assignment is weighted by the probability of each “neighbor” being correct, and the probabilities at stage *m* can be updated by replacing them by the new weight configuration state. As probabilities evolve, the information content of changing configurations is monitored for the optimally “informative” state. The resulting model is analogous to the random cluster Fortuin and Kasteleyn (FK) model [43]. In practice, a straightforward implementation leads to densely connected networks with noisy weights and no principled way to control the iteration steps.

To implement the intuitively appealing ideas presented above that are designed to find the optimal state in the form of marginal probabilities, we have devised an iterative approach that utilizes topology selection followed by a variation of belief propagation algorithm [42] and subsequent adjustment of initial weights and topology. This topology selection step plays a key role in achieving robust and computationally efficient results.

### Mathematical Formulation

We proceed by analogy to FK [43]. Let *G* = (*V*,*E*) be any general graph, with *e*∈*E* an edge in *G*, and *ν∈V* a vertex. The set of assignments (or labels) for each vertex is designated by [1,2,…,*q*]. The “configuration energy” of this system is encoded in the partition function:
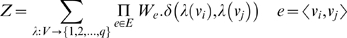
(1)


In this formula, the outside sum is performed over the configuration states of the system represented by the map λ, and the inside product measures the compatibility of the vertex labels joined by the edge *e*. Each edge is weighted by the factor 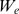 and has end-point vertices 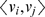, and δ is the compatibility measure of end-point vertices configuration. By defining 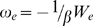 and 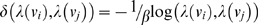, Eq 1 can be rewritten as:
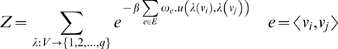
(2)


In the setting of statistical physics, the Boltzmann weight of a configuration is 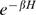, where *H* (the sum in the exponential) represents the energy of the configuration and *β* is a parameter called the inverse temperature. Because the weights are assumed to be positive, they can be interpreted probabilistically (after normalization by *Z*) as a probability measure on the 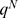 states for the graph *G* where *N* is the number of vertices.

In the standard random-cluster model, the neighborhood structure, or topology, of the graph is prescribed, and the objective is to find the ground state for a given set of weights by varying the “spin”, or labeling, configurations. In our case, we are determining the ground state ensemble and the topology of the model at the same time. At each iteration step *i*, we define *A_i_*, a subset of the graph G, where 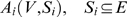, and evaluate the partition function for this subset. We evolve the topology of the graph at each iteration by the addition and removal of edges and by refining the edge weights toward the optimum topology as described in the algorithm section. A local Bayesian updating procedure updates the weights, and the local rate of change of weights is used to modify the corresponding local topology of the graph. On the subsequent iteration, our algorithm reintegrates these local modifications in the context of the entire network and attempts to establish a new quasi-stationary state.

The algorithm must address two critical challenges. The data that describe edge weights and states in Eq 2 are derived from empirical relationships that involve noisy data, and, therefore, a straightforward deterministic search of the resulting combinatorial space would be infeasible. In addition, the computational complexity of the resulting problem grows rapidly with the number of labels and the topology of the graph; thus, a suitable starting and evolving representation of the topology, and a corresponding approximation algorithm is the key to obtaining a robust solution to this problem.

### PINE-NMR

The probabilistic construction used in PINE-NMR belongs to the general class of graphical models in which dependencies among random variables are constructed ahead of the inference task. In cases where the graph of dependencies is acyclic, there are powerful and efficient algorithms that correctly maximize the marginal probabilities through collecting messages from all leaf nodes at a root node [44]. When the graph is not acyclic, current algorithms for graphs with cycles often reach oscillatory states, converge to local maxima, or achieve incorrect marginals due to computational difficulties. Approaches have been described in the literature for dealing with a single loop condition [45] or for converging under alternative free energy approximations [46],[47]. “Tree-based reparameterization” algorithms [48] have been described as a general approach that iteratively reparameterizes the distributions without changing them on the subtrees in the original graph. These algorithms, which are geared toward addressing the approximation of marginals in the presence of loops, represent trade-offs among robustness, accuracy, computational speed, and efficiency of implementation. Our modification provides a simple extension that can be described as an adaptive form of coarse-to-fine approximation. We start with a “coarser topology” and explore more refined factorizations of the probability distribution and look for stable fixed points. In our adaptive approach, the extension of the state space (embodied in the algorithm) plays a critical role. In intuitive terms, the additional degrees of freedom (null states) provide “room for change” for existing distributions as the topology is being refined. The internal working of the stepwise factorization of the probability distribution requires a coarse estimate on the initial threshold that reduces the connectivity degree of the graph. In our case, this approximation is arrived at using a combination of theory and empirical investigation.

**Figure 4:**
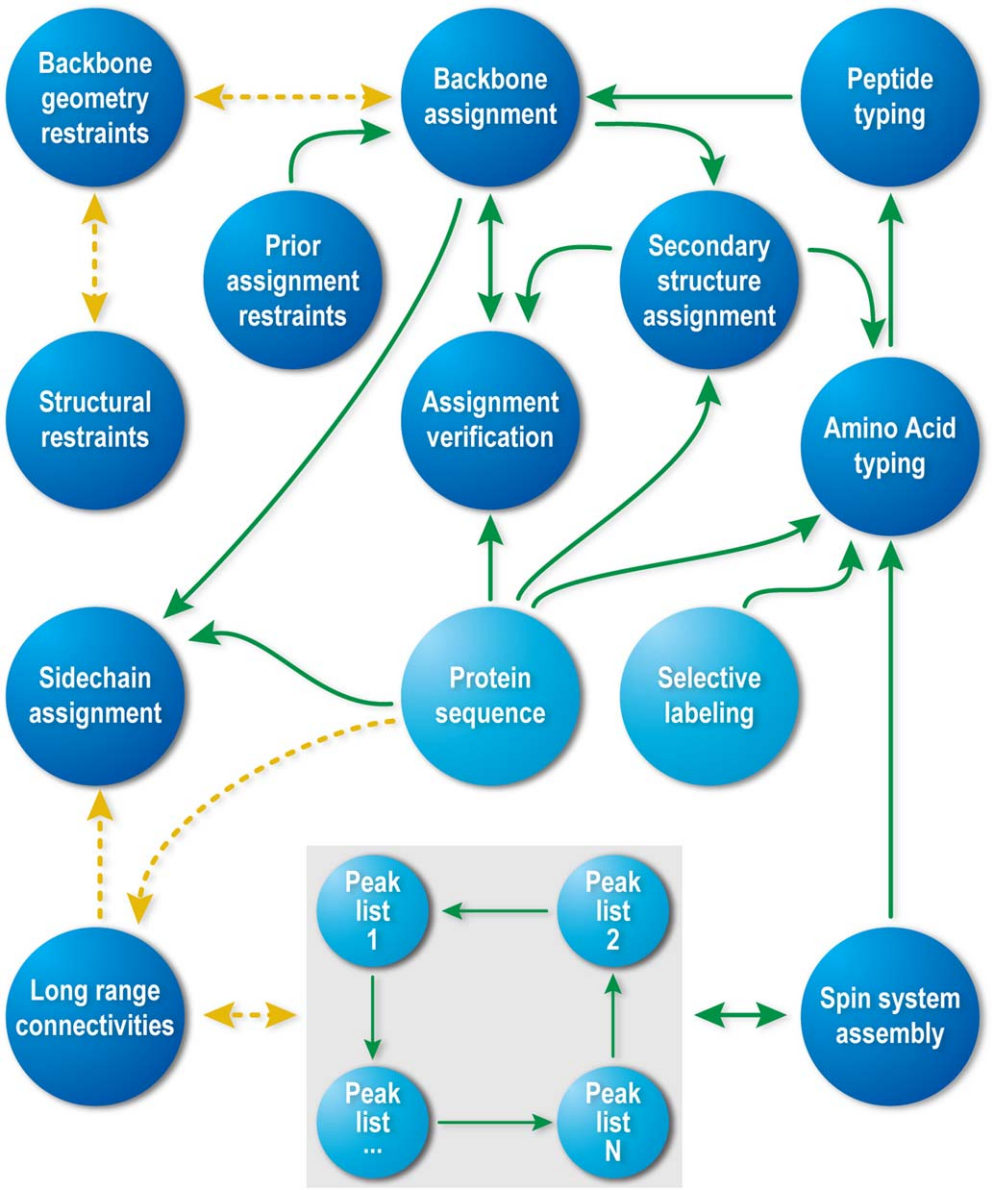
Global network of relationships in PINE-NMR. A set of probabilistic influence sub-networks are combined into a larger influence network. The iterative probabilistic inference on the complex network ensures globally consistent labeling.

Figure 4 presents an overview of the probabilistic network implemented in PINE-NMR. Sets of probabilistic influence sub-networks are combined into a larger influence network, and each sub-network may have its own computational model used to perform the inference task. The entire probabilistic network is constructed by considering the conditional dependencies of the sub-networks. The actual implementation of PINE-NMR entails a fairly complicated network with more than 30,000 lines of code in Matlab and other supporting scripting language. A descriptive and stepwise version is given below.

### Basic Algorithm for PINE-NMR

**Figure 5:**
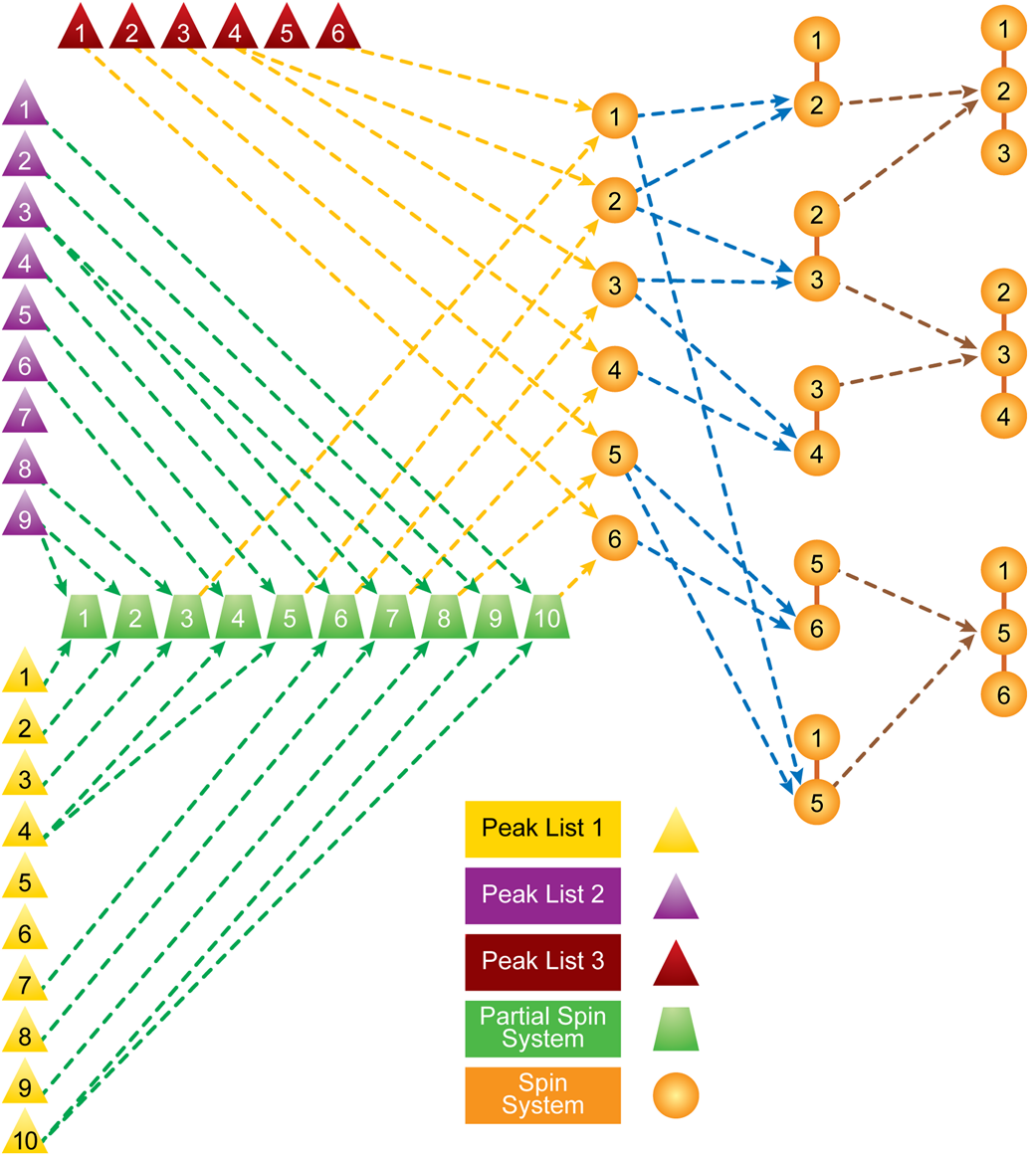
Spin system generation network in PINE-NMR. The peaks in the most sensitive experiments in the data are used initially as reference peaks. Aligning the peaks along the common dimensions and registering them with respect to reference peaks enables us to define a common putative object called the spin system. Spin systems are then assembled to derive triplet spin systems.

1. Read input data and check for errors. If errors are found, report errors and abort.

2. Align the ^1^H, ^15^N, and ^13^C dimensions of all spectra independently.

3. Generate spin systems (Figure 5).

- Derive the similarity scores of peaks *X^i^_j_* across spectra.
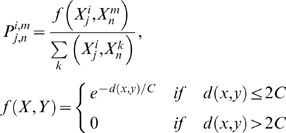
(3)The distance function denoted by *d* is the ordinary Euclidean distance along the common dimensions of the vectors, and *C* is determined by the spectral resolution in each dimension of a multidimensional experiment. The default values are 0.25 ppm for ^15^N and ^13^C, and 0.02 ppm for ^1^H. The existence of peaks closer than default values in any spectra adjusts *C* accordingly. The values can also be overridden manually.
- Begin with sensitive spectra; build probabilistic spin systems for backbone atoms:
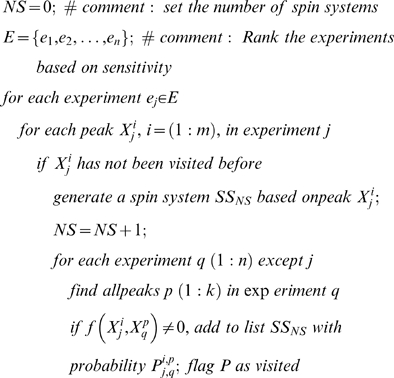

- The resulting spin systems have the following fields (some fields might be empty or have several choices with different probabilities):
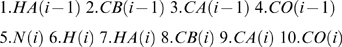

- Derive connectivity scores for spin systems by a formula analogous to 3.a. The score *P(SS_i_,SS_j_)* is measured using fields *7–10* of *SS_i_* and fields *1–4* of *SS_j_*.
- Utilize the scores to assemble the spin systems to triplet spin systems.

4. Estimate the *b factor* and *c factor*, which are the measures of data quality defined as follows:
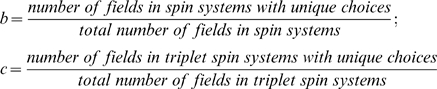
In calculating any of the above formulas, only the fields with choices are considered. For example if none of the experiments provided by the user has HA information, HA fields are not used in the calculation.

5. If *(b<0.4 or c factor<0.2)* # comment: Report low data quality to the user and stop. The low data quality check can be manually overridden through user requests. However, low “quality factors” are strong indicators of “highly incomplete” data and the web service discourages the use of results from low quality data.

6. Otherwise, set *K = 0* (iteration counter).

**Figure 6:**
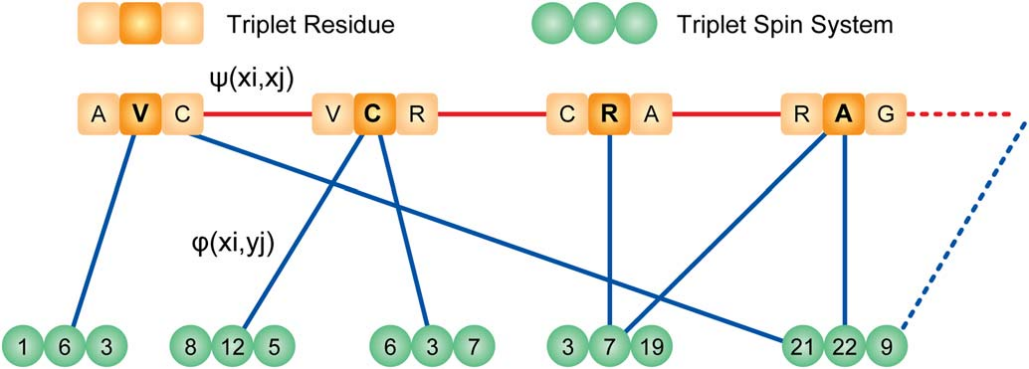
Graphical network for backbone chemical shift assignments. Overlapping tripeptides (triplet residue) are evaluated. The weights on the edges are derived from amino acid typing, secondary structures, connectivity experiments, and possible outlier assignments. According to the statistical physics model described in the text, application of the belief propagation algorithm yields the marginal probabilities for backbone assignments.

**Repeat**:

7. *K = K+1*; (iteration counter).

8. Triplet amino acid typing:

- Score each atom based on the probability distribution of chemical shifts derived from BMRB, and the latest secondary structure prediction.
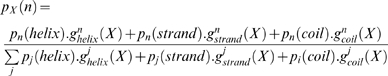
(4)

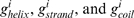
, are the chemical shift probabilities of the related atom in residue *i* derived from BMRB and PDB databases, and *p_i_(helix)*, *p_i_(strand)*, and *p_i_(coil)* are the secondary structure probabilities in the current iteration step.
- Adjust the scoring if any pre-assignment exists:
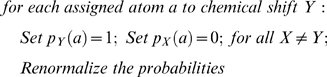

- Adjust the scoring if any selective labeling experiment exists while taking into account the possibility of overlap:
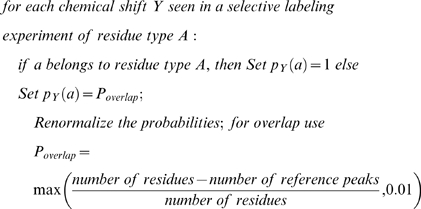
We set the minimum probability of a peak to possibly overlap with another one to 0.01, even if the number of peaks in the dataset exceeds the number of residues.
- The total score S*x(y)* of labeling a triplet spin system *y* to a triplet residue *x* is the product of scores of individual atoms in the triplet residue. Both triplet spin systems and triplet residues contain an extra state called the “null” state to allow for the case where they cannot be labeled.

9. Derive the backbone assignment network weights based on amino acid typing scoring, connectivity experiments, latest backbone assignment, and possible outlier detections from the last iteration (Figure 6):
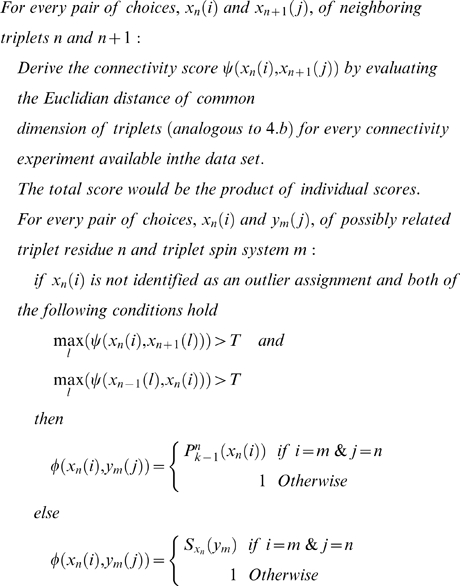

*T* is a threshold value for the connectivity score, which is defined as, c*max_connectivity_score, *c* is the quality factor defined in *5*, and *P_k-1_(x_n_(i))* is the probability of assigning *x_n_(i)* to triplet residue *n* in the iteration *k−1*.

10. Select the network topology; calculate the threshold for removing low-weight edges from the network based on the quality of the data, use: 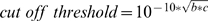


11. Apply the belief propagation algorithm [42] to find the marginal probabilities *P_k_^n^(x_n_(j))* of assigning triplet spin systems *x_n_(j)* to triplet (tripeptide) residues *n*.

12. Given the marginal probabilities of the triplet residue assignments, derive the probabilistic assignment of the individual backbone atoms.

13. Detect and remove the outliers in the backbone assignments [16].

14. Derive the secondary structure of each amino acid based on the formula: 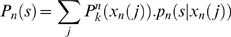
(5)
*p_n_(s|x_n_(j))* is the probability of residue *n* to be in the secondary structure state *s* given the assignment *x_n_(j)* derived from the method described in [13], and *P_k_^n^(x_n_(j))* is the assignment probability of triplet residue with the center residue *n*, to triplet spin system *x_n_(j)*. The summation is over all the possible choices of tripeptides in the protein sequence.

### Until the assignment probabilities converge or K = 10 (the maximum number of iterations)

15. If no convergence, probabilities are the average probability of last three iterations. “No convergence” indicates the presence of “nearby” local minima.

16. For every amino acid, generate an energetic model network and apply the Belief Propagation [42] to derive final probabilistic side chain assignments as described in supplementary material Protocol S1.

17. Report the final probabilistic assignments: backbone, side chain, secondary structure prediction, and possible outliers. The output can be specified to conform to variety of formats, including Xeasy, SPARKY, and NMR-STAR (BMRB).

The input to PINE-NMR consists of the amino acid sequence and multiple datasets known as peak lists (chemical shifts) obtained separately from selected, defined NMR experiments. The peak lists consist of sets of real-valued two-dimensional, three-dimensional, or four-dimensional vectors, denoted by *^l^X* ^i^_j_∈*R^l^ l = 2,3,4*. The dimension of the data is denoted by *l*, the index *j* indicates that the observation is from the *j* ^th^ dataset, and the index *i* denotes the *i* ^th^ observation within the dataset. To compare data from different experimental sets (different *j*) that have shared subspaces (signals from nuclei in common), we consider only the common subspace. This allows us to omit the index *l* in subsequent formulas. The similarity (or nearness) is used to build an initial system of neighborhoods. The approximate starting value for similarity is given a probabilistic interpretation by using Eq 3 (Basic Algorithm: 3.a) to compare each datum (peak) *X^i^_j_* with the reference datum (peak) *X^m^_n_*. The peaks in the most sensitive experiments in the dataset (normally ^15^N-HSQC or HNCO) are used as the initial reference set. We define a common putative object, called the spin system (Figure 6), by aligning the peaks along the common dimensions and by registering them with respect to reference peaks according to Eq 3. The total number of states of the spin system is equal to the combinatorial set of all label choices including the null state. The preservation of all neighborhood information at this step is particularly important for the analysis of data from larger proteins in which noise peaks and real peaks are closely interspersed.

### Amino Acid Typing (Spin System Scoring)

The spin-system scoring step is used to integrate the spin system sub networks by assigning a score to each possible label that can be associated to a spin system. This process makes use of empirical chemical shift probability density functions, calculated from combined BMRB (chemical shift) and PDB (coordinate) data from proteins of known structure, for each atom of every amino acid type in three label states: α-helix, β-strand, and neither helix nor strand (other) [13]. The general form of the score is obtained by computing the probability of a chemical shift *X* having the label *n* (residue number) as described in Basic Algorithm: 8.a. This approach connects amino acid typing and secondary structure state determination through a conditional dependency model. The successive application of weighted measures (Basic Algorithm), leads to the definition of a complex network of relationships and weights among correlated sets of information at the global level (Figure 3). This process establishes an initial system of neighborhoods (Figure 2). Whenever an initial set of probabilities is unavailable, a uniform distribution is assumed as the starting state.

### Backbone and Side Chain Assignments

The challenge is to address the computationally demanding problem of deriving the backbone and side chain assignments from amino acid typing and other experimental data (connectivity experiments) according to the model described above. Rather than modeling the assignment of labels to individual peaks, or assigning spin systems to a single amino acid, we generate triplet spin systems and label them to overlapping triplets of amino acids in the protein sequence (Figure 5). The selection of tripeptides instead of single residues reduces the complexity of the graph by eliminating a substantial number of labeling choices; however, it may introduce additional noise to the network due to possible erroneous spin system assembly. Given the trade-off between noise level and network complexity, we found that triplets yielded the optimum choice among other combinations of residues. However, the resulting network of weights and relationships has a complex topology in which a large fraction of relationships (edges) arise entirely from noise in the data, and the resulting random field is not amenable to a straightforward implementation. To overcome this problem, we determine, from spin system scoring and connectivity constraints, an initial topology and the sets of weights for the backbone (Figure 6 and Basic Algorithm: 9) and side chain assignments (Protocol S1). The topology ordinarily is dependent on the weights and a set of parameters (thresholds). These values typically are noisy and incomplete and are contaminated by false positives and false negatives. Our goal is to evolve the initial state of the system toward an “optimally coherent” state without the need for any manual parameter settings by carefully managing the selection of network topology. An initial topology for the network is determined by removing all edges with potential weights below a threshold value. The threshold value is calculated (Basic Algorithm: 10) automatically by approximating the level of success achievable by each threshold (Figure S1). For a fixed set of edge values, this function is generally unimodal and defines the appropriate threshold for the starting state. At each threshold, a variation of the belief propagation algorithm [42] operates on the dense multigraph to effectively prune many edges and to derive the posterior probabilities that define clusters (or labels). After each iteration step, the posterior probabilities of all label assignments are utilized to determine local topology modifications and new edge weights.

### Assignment of Secondary Structure Labels

Secondary structure labels are dependent variables derived from prior chemical shift assignments. Each chemical shift assignment has an associated probability, and we derive the probabilities for the assignment of secondary structure labels from a normalized and weighted sum of associated probabilities. After computing the probability of each residue *n* to be in each of three conformational states (*s* = helix, strand, other) by the method described in [13] for different assignment configurations, the overall secondary structure probability is calculated by Eq 5 (Basic Algorithm). Note that this step involves a shift in the point of view from chemical shift centric to residue centric.

### Iteration Rules

Posterior probabilities derived in each iteration of the assignment process are used as local prior probabilities in the next round of assignment, provided that (1) the assignment has not been detected as an outlier, (2) the assignment of chemical shift is correlated with the assignment of secondary structure consistent with known empirical distributions, and (3) the assignment is consistent with established connectivity constraints.

If one or more of the above conditions are not met, the results are deemed inconsistent because the resulting probabilities appear as outliers of the marginals supported by the current graph topology. This view is driven by the notion that the equilibrium of our fictitious system is the fixed point of the energy functional, with the factorization induced by our graph. In order to reach the consistent state, scores are re-evaluated and a new local score is computed for the next iteration; a new topology is generated, and the computational steps are repeated. The iteration process continues until a stationary or quasi-stationary state is reached, i.e., when the topology of the network and the labeling probabilities do not vary significantly. The iteration process leads to “self-correction” through appropriate adjustments to the topology of the underlying network in order to preserve maximum information.

## Results

PINE-NMR is designed to analyze peak lists derived from one or more of a large set of NMR experiments commonly used by protein NMR spectroscopists. This set (listed on the PINE-NMR website) currently includes data types used for backbone and aliphatic side chain assignments. (PINE-NMR will be expanded in the future to handle aromatic side chain assignment.) To test the software, we asked colleagues at the Center for Eukaryotic Structural Genomics (CESG) and the National Magnetic Resonance Facility at Madison (NMRFAM) to provide subsets of data from projects that had led to structure determinations with assigned chemical shifts deposited in the BMRB [49]. We wanted the assignments to have been refined and vetted in light of a structure determination, because we took the BMRB deposited values to be “correct”. In most cases, the input data supported the determination of both backbone and aliphatic side chain assignments. In some cases, the input data supplied supported only the determination of backbone assignments. The peak lists were provided by the persons submitting the data without any specification for the peak picking software, threshold, or other parameters.

Table 1 summarizes the PINE-NMR results for all datasets provided. The input datasets are indicated along with the size of the protein. A backbone or side chain assignment was scored as “correct” if the top ranked (highest probability) PINE-NMR assignment corresponded that in the BMRB deposition. The assignment accuracy is given as the number of “correct” assignments divided by the total number of assignments supported in theory by the input data expressed as a percentage. “The “correct” (BMRB) assignments had the benefit of additional information coming from NOESY data and filtering with respect to structure determination. Also listed in Table 1 is the backbone “assignment coverage” achieved by PINE-NMR (defined as the total number of correct backbone assignments in comparison to the total backbone assignments in the corresponding BMRB deposition expressed as a percentage).

The secondary structure accuracy reported in Table 1 compares the PINE-NMR result with the secondary structure of the deposited three-dimensional structure as determined by the DSSP software [50]. It can be seen that the accuracy of the PINE-NMR results correlates with the data quality factor. The outlier count is defined as the number of C′, C^α^, or C^β^ atoms detected as possible outliers in the final assignment by the LACS method [16].

In the majority of cases, the assignment accuracy was above 90% for backbone resonances and above 80% for aliphatic side chain resonances. Two cases in Table 1 yielded assignment accuracies below 90%. In the case of the 177-residue protein (At5g01610), the lower performance was due to the poor quality of data from a highly disordered region of the protein. A human expert was unable to go beyond the PINE-NMR assignments, and additional data were required to complete the protein structure determination. In the case of the 299-residue protein (At3g16450), its stereo array isotope labeling (SAIL) pattern [51] gave rise to chemical shift deviations that degraded expected matches. In this case the performance of PINE-NMR could be improved by incorporating corrections for the deuterium isotope effects on the chemical shifts.

An illustration of the improvement achieved by combining information comes from comparing the assignment accuracy results from PINE with those from PISTACHIO [12] (Table 1). PISTACHIO is an automated assignment tool developed earlier that does not make use of inferred secondary structure or outlier detection implemented in PINE-NMR. The results from PINE-NMR also are superior to those achieved by iterative pipelining of the individual assignment (PISTACHIO [12]), secondary structure determination (PECAN [13]), and outlier detection (LACS [16]) steps (results not shown). The tests of PINE-NMR shown in Table 1 are highly stringent, in that minimal information is provided. Separate tests (results not shown) demonstrate that the performance is improved if the input peak lists have been pre-filtered to correspond to spin systems.

The results of website users provide a separate measure of the performance of PINE-NMR. Since July, 2006, users have analyzed more than 1,300 sets of chemical shift data. Without access to the final structures and chemical shift assignments for these proteins, these results could not be analyzed, as in Table 1, with regard to correct assignments and secondary structure. Instead, we used the results from Table 1 to estimate the empirical conditional probability of incorrect labeling in the user PINE-NMR output: *P(incorrect label| p_label_ = x)*. Assignments with a probability higher than 0.95 generally were found to be correct (Table 1). Using the data submitted to the PINE-NMR web site, we selected a representative sample of proteins with numbers of residues and data quality factors similar to those in Table 1. We then used the empirical estimate of accuracy to analyze the results from these proteins (Table S1). The outcome was in substantial agreement (in a statistical sense) with the results shown in Table 1. Of particular note are two proteins submitted to PINE twice (the proteins with 120 residues and 160 residues in Table S1). In each case, after an initial submission of the data, the user provided additional experimental data prior to another round of analysis. The additional data improved the empirical estimate of accuracy and led to additional assignments at improved levels of confidence.

The level of accuracy and completeness achieved in favorable cases by a single automatic PINE-NMR computation was sufficient for the initial downstream steps of structure determination. For example, the PINE assignment output for ubiquitin, which was obtained from the input of automatically picked peak lists from HSQC, HNCO, CBCA(CO)NH, HNCACB, C(CO)NH, H(CCO)NH, HCCH-TOCSY, HBHA(CO)NH, and C13-HSQC spectra, along with ^15^N-NOESY and ^13^C-NOESY spectra for this protein were provided as input to the Atnos [52]/Candid [53] program. The only manual step in the structure calculation was the determination of cross β-strand hydrogen bond constraints for the amino acid residues shown to be in β-sheet by the PINE analysis of secondary structure (an effort taking only about one hour). Hydrogen bond constraints for α-helical regions were introduced based on the results of the PINE secondary structure analysis. The resulting 20 conformers that best fit the input data had an rmsd of 1.1 Å for backbone atoms and 1.7 Å for all heavy atoms (0.8 Å for backbone residues and 1.3 Å for all heavy atoms in ordered residues as analyzed by PSVS [54]. This structure had a backbone rmsd of 1.23 Å from the highly refined ubiquitin structure determined from NMR data deposited in the PDB (1d3z). Without the manual hydrogen bond constraints the structure had a backbone rmsd of 2.77 Å from the 1d3z structure.

The level of assignments achieved by PINE-NMR for small proteins meets or exceeds the assignment levels that led to successful structure determination of small (under 100 residue) proteins from chemical shift data alone [5].

PINE-NMR also can be useful for semi-automated analysis of larger proteins that require for structure determination the collection of additional data such as dipolar couplings, manual NOESY assignments, or aromatic side chain assignments. We have developed PINE-NMR in ways that enable expert input, for example, by specifying a selective labeling scheme, pre-assigned cluster labels, pre-assigned spin systems, or pre-assigned cluster labels for subsets of the data. For pre-assigned cluster labels, PINE-NMR can act as a verification tool, for example, by checking their internal consistency with peak lists or by detecting chemical shift referencing problems or outliers (the LACS report). The software performs spectral alignment, detects excessive noise peaks, uncovers experimental inconsistencies, recognizes the insufficiency of input data, and identifies nomenclature conflicts.

The latest version of PINE-NMR is available for public use through a webserver at http://pine.nmrfam.wisc.edu. The PINE-NMR server offers complete backbone and side chain chemical shift assignment, secondary structure determination, and possible referencing error or outlier detection. The server supports a variety of convenient input and output formats, including Sparky, Xeasy, and BMRB (NMR-STAR). PINE-NMR also accepts prior information that reflects experimental information the user wishes to specify, such as fixed input (pre-assigned labels), selective labeling pattern, or assembled spin systems in cases where segments of the protein have been labeled by other means.

## Discussion

Application of the PINE algorithm to the NMR assignment problem has led to a tool that is capable of analyzing data in a self-correcting manner without the need for the user to manipulate any parameters in the software. The public availability of PINE-NMR through an online server has made it possible for a variety of users to test its accuracy and robustness. The PINE algorithm reformulates an otherwise intractable network of interactions within the context of an energy minimization problem. To address the high computational complexity of the minimization problem, we have devised a local approximation algorithm with reliable global properties. To address the non-convexity of the energy functional and the potential of “getting stuck” in local minima, we perform successive approximations with increasingly more complex energy functionals and with the reweighting of solutions.

Our evolution and selection of the initial network topology of PINE-NMR emerged through the examination of two quantities: (1) the estimated conformity across all datasets with respect to a single reference dataset (*b* factor), and (2) the estimated conformity between pairs of datasets that contained complementary information (*c* factor). These quantities, which are calculated as described in the Basic Algorithm, were found to be generally dependent on the size of the protein and the number of false positive and false negatives in the input data. In intuitive terms, the combination of these quantities measures the degree of conformity between the vertex and edge potentials in the network model. The numerical approximation of this quantity (in analogy to quantity called a matching polynomial) is encoded in the fourth root of the product of *b* and *c*. For example, when pairs of data in the dataset have low conformity measures, the network topology (e.g. change in the edge set) is strongly influenced by label assignments. These same quantities are also used in the computation of the quality factor and the predicted number of residues assigned (Table S1). After a user submits input data to the server, PINE-NMR performs a preliminary evaluation. If factors *b* and *c* do not satisfy the required threshold, PINE reports the problem to the user and suggests possible remedies. Otherwise the assignment process continues. Typically the datasets that yielded high-quality assignments in PINE-NMR had *b* factors equal to 0.65–0.85 and *c* factors equal to 0.4–0.6.

The impact of topology selection can be investigated computationally by running simulations that test the computational complexity (running time) and accuracy of the results as a function of increasing network complexity. For small proteins, where the number of false positives and negatives is small, increasing network complexity leads asymptotically to higher accuracy (Figure S1A). The network energy remains stable as more edges are added, and the computational complexity drops sharply as soon as an “essential network topology” is achieved. For larger proteins, increasing network complexity initially leads to higher accuracy, but accuracy falls off at the highest levels of complexity (Figure S1B). The most accurate label assignments are achieved when the cardinality of the edge set for the network is small. Therefore, selecting a more complex network of interactions not only is computationally inefficient but may also lead to decreased accuracy. Inaccuracies within more complex networks tend to propagate. Specifically, high complexity neighborhoods with large numbers of edges were found to degrade the accuracy of their neighbors, and, although this effect typically is local, it also can have long-range impact. These findings reinforce the importance of selecting good initial topology and underscore the advantages of local, as opposed to global, topology modification.

In practical terms, additional knowledge about the structure of a protein can improve the data interpretation. For example, NMR experts often use their experience and knowledge of similar structures or structural folds to make decisions – this knowledge is often hard to codify in an algorithm. In some instances, the bias is subtle. For example, the use of data from BMRB in order to generate simulated peaklists that are to be subsequently assigned is afflicted with bias, because the data in BRMB are highly likely to be associated with a known structure and, therefore, higher information content (sharper localization of parameters according to Bayes' formula).

One of the challenges in protein NMR spectroscopy is to minimize the time required for multidimensional data collection and analysis without sacrificing the quality of the resulting protein structure. We are in the process of coupling PINE-NMR to (HIFI-NMR) [55], an innovative approach that uses adaptive reduced dimensionality NMR data collection. For 3D triple-resonance experiments of the kind used to assign protein backbone and side chain resonances, the probabilistic algorithm used by HIFI-NMR automatically extracts the positions (chemical shifts) of peaks with considerable time-savings compared with conventional stepwise approaches to data collection, processing, and peak picking. The combination of HIFI- and PINE-NMR will support fully automated, probabilistic, NMR data collection and analysis through assignments, determination of secondary structure and backbone dihedral angles. We are currently developing protocols for including H(C)CH-COSY, CCH-TOCSY and common four dimensional NMR experiments in the PINE-NMR network. Our future plans also include the inclusion of NOESY data, which will extend side chain assignments to aromatic residues [56] and support assignments of larger proteins [3].

The core computational model of PINE should be applicable to other problems where automated clustering is needed. For example, when DNA microarray data are used to explore all genes of an organism in order to detail their biochemical networks, automated clustering of gene networks can provide unbiased information about the underlying biology.

## Supporting Information

Figure S1 Running time and assignment accuracy of the results as a function of increasing network complexity. Network complexity is defined as: network complexity = −log(cutoff threshold). The results for smaller proteins or proteins with higher quality data (A) differ from those for larger proteins with low quality data (B). The results underscore the importance of proper setting the cut-off threshold in selecting the edge set when constructing the topology of the graph.
(0.09 MB TIF)

Protocol S1 Side chain chemical shift assignment algorithm.
(0.03 MB DOC)

Table S1 Examples of ten PINE-NMR runs with experimental NMR data showing how the data quality measure *t* correlates with the agreement between the actual and predicted number of assignments with probability *p*>0.95. The strong correlation can be best observed in the cases where additional data for the same protein have been uploaded to the server.
(0.23 MB DOC)
