# Supplementary figures and images for: Probabilistic Interaction Network of Evidence Algorithm and its Application to Complete Labeling of Peak Lists from Protein NMR Spectroscopy
Source: PLoS Comput Biol. 2009 Mar 13;5(3):e1000307. doi: 10.1371/journal.pcbi.1000307 (PMC2645676; doi:10.1371/journal.pcbi.1000307)

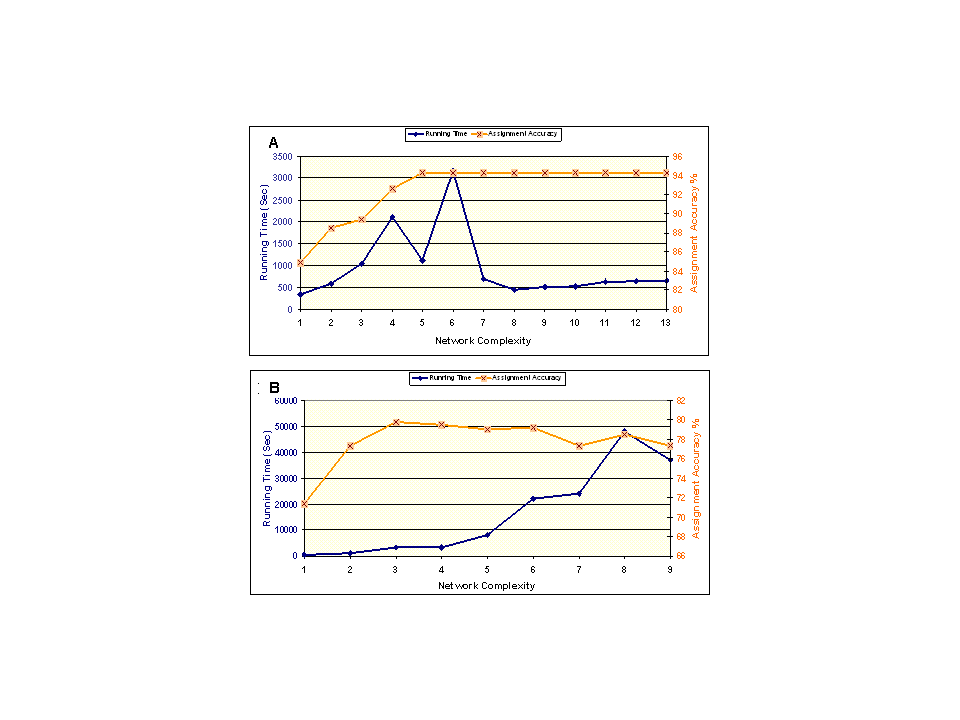

Supplement: Figure S1 — Running time and assignment accuracy of the results as a function of increasing network complexity. Network complexity is defined as: network complexity = −log(cutoff threshold). The results for smaller proteins or proteins with higher quality data (A) differ from those for larger proteins with low quality data (B). The results underscore the importance of proper setting the cut-off threshold in selecting the edge set when constructing the topology of the graph. (0.09 MB TIF) [file pcbi.1000307.s001.tif]
