# Supplementary material for: Probabilistic Interaction Network of Evidence Algorithm and its Application to Complete Labeling of Peak Lists from Protein NMR Spectroscopy
Source: PLoS Comput Biol. 2009 Mar 13;5(3):e1000307. doi: 10.1371/journal.pcbi.1000307 (PMC2645676; doi:10.1371/journal.pcbi.1000307)
Supplement: Protocol S1 — Side chain chemical shift assignment algorithm. (0.03 MB DOC) [file pcbi.1000307.s002.doc]

**Protocol S1.** Side chain chemical shift assignment algorithm.

1. For each side chain atom *X^i^_j_* in residue *j* of the protein derive the possible assignment candidates as follows:

*Calculate the prior chemical shift probability distribution from BMRB data: P_prior_(X^i^_j_)*

*For every peak k in the whole side chain data set with chemical shift vector (k_1_, k_2_,…,k_n_) in each dimension n and for every corresponding side chain atom X^i^_j_ apply Bayes’s rule:*

$P_{posterior}(X^{i}_{j}=k_{l}|\exists Peak_{k})=\frac{P_{prior}(X^{i}_{j}=k_{l})*P(\exists Peak_{k}|X^{i}_{j}=k_{l})}{P(\exists Peak_{k})}$

where *k_l_* is the chemical shift value of peak *k* in dimension *l*, and $\exists Peak_{k}$denotes the probabilistic event of observing peak *k* in spectra. The expression $P(\exists Peak_{k}|X^{i}_{j}=k_{l})$ represents the probability of observing peak *k* in the spectra given that the chemical shift of atom *X^i^_j_* is *k_l_*. The estimate uses a model that is specifically built for each experiment. For example, with data from an H(CCO)NH experiment (or other side chain experiment that includes the amide group), this probability would simply be the probability that the chemical shift assignments of the amide nitrogen and amide proton of residue *j* match the chemical shifts of peak *k* (backbone assignment probabilities). Note that the estimates for the probability, as well as estimates for the normalization factors, are not required to be precise. These posterior estimates based on Bayes’ formula merely provide the initial values for the energy function described in the section below.

*Renormalize $P_{posterior}(X^{i}_{j}|\exists Peak_{k})$ and Set P_prior_(X^i^_j_) to* $P_{posterior}(X^{i}_{j}|\exists Peak_{k})$

2. For each side chain atom *X^i^_j_* extract the top *n* assignment candidates with the highest *P_prior_(X^i^_j_)*, namely {*X^i^_j_(1)*, *X^i^_j_(2), …, X^i^_j_(10)* }. The value of *n* = 10 is the default value (a constant) deemed to be sufficiently large for capturing all candidates.

3. Set up a separate network model for the side chain assignment of each residue *j* of the protein. Establish the set of vertices as *V* *={X^1^_j_, X^2^_j_, …, X^n^_j_}*. Define the partition function of the system as follows:

$$Z=\sum_{\lambda:V\to\{1,2,...,10\}} e^{-\beta(\sum_{i,j,k} u_{3}(\lambda(v_{i}),\lambda(v_{j}),\lambda(v_{k})) +\sum_{i,j} u_{2}(\lambda(v_{i}),\lambda(v_{j})))}$$

The Hamiltonian (energy function) of the system has two terms *u_3_* and *u_2_*. The summation is over all combination of triplets and pairs of atoms in residue *j*. The *u_3_* terms are derived from the peak lists from three-dimensional NMR experiments. In intuitive terms, these values measure how probable the configuration is given the peak lists in relation to a distance function similar to that in Eq 3. The *u_2_* terms account for peak lists from two-dimensional NMR experiments such as ^13^C-HSQC. The idea can be readily extended to data from four-dimensional NMR experiments.

4. Derive the marginal probabilities of the side chain atom assignments by applying the belief propagation algorithm [1].

Reference

1. Yedidia JS, Freeman WT, Weiss Y (2005) Constructing free-energy approximations and generalized belief propagation algorithms. Ieee Transactions on Information Theory 51: 2282-2312.
