# Supplementary material for: Probabilistic Interaction Network of Evidence Algorithm and its Application to Complete Labeling of Peak Lists from Protein NMR Spectroscopy
Source: PLoS Comput Biol. 2009 Mar 13;5(3):e1000307. doi: 10.1371/journal.pcbi.1000307 (PMC2645676; doi:10.1371/journal.pcbi.1000307)
Supplement: Table S1 — Examples of ten PINE-NMR runs with experimental NMR data showing how the data quality measure t correlates with the agreement between the actual and predicted number of assignments with probability p>0.95. The strong correlation can be best observed in the cases where additional data for the same protein have been uploaded to the server. (0.23 MB DOC) [file pcbi.1000307.s003.doc]

**Table S1.** Examples of ten PINE-NMR runs with experimental NMR data showing how the data quality measure *t* correlates with the agreement between the actual and predicted number of assignments with probability *p* > 0.95. The strong correlation can be best observed in the cases where additional data for the same protein have been uploaded to the server.

| PINE ID | Number of residues (assignable residues) ^a^ | Number of residues (percent) assigned with *p* > 0.95 | Number of residues (percent) assigned with *p* > 0.75 | Percent  assigned  secondary structure | Outlier  count | Quality measure ^b^ | Predicted number of residues assigned with *p* > 0.95 ^c^ |
| --- | --- | --- | --- | --- | --- | --- | --- |
| 34375870 | 90 (79) | 74 (94%) | 77 (97%) | 84% | 4 | 0.94 | 74.2 |
| 84652704 | 92 (86) | 83(97%) | 84 (97%) | 84% | 1 | 0.94 | 80.4 |
| 42246521 | 120 (116) | 95 (82%) | 96 (83%) | 87% | 1 | 0.97 | 96.4 |
| 72660091 | 120 (116) | 99 (85%) | 99 (89%) | 87% | 1 | 1.00 | 100.6 |
| 78060302 | 124 (116) | 97 (84%) | 110 (94%) | 85% | 2 | 0.70 | 81.0 |
| 71866627 | 130 (120) | 98 (82%) | 116 (95%) | 92% | 1 | 0.82 | 98.0 |
| 17115465 | 151(137) | 122 (89%) | 126 (92%) | 86% | 1 | 0.79 | 108.5 |
| 41013430 | 160 (149) | 126 (85%) | 142 (95%) | 81% | 4 | 0.84 | 123.7 |
| 77778813 | 160 (149) | 141 (95%) | 143 (96%) | 82% | 1 | 0.91 | 135.1 |
| 61203202 | 174 (167) | 120 (72%) | 124 (75%) | 86% | 1 | 0.85 | 142.5 |
| a. Excluding proline residues and histidine tags.  b From the equation: , where, aa = number of amino acids; ss = number of spin systems; b = *b factor* consistency measure; c = *c factor* consistency measure.  c. From the equation: , where, aa = number of amino acids; ss = number of spin systems; b = *b factor* consistency measure; c = *c factor* consistency measure. | | | | | | | |
